# Soft Character of Star-Like Polymer Melts: From Linear-Like Chains to Impenetrable Nanoparticles

**DOI:** 10.1021/acs.nanolett.2c04213

**Published:** 2023-01-09

**Authors:** Petra Bačová, Eirini Gkolfi, Vagelis Harmandaris

**Affiliations:** † Departamento de Ciencia de los Materiales e Ingeniería Metalúrgica y Química Inorgánica, Facultad de Ciencias, IMEYMAT, Campus Universitario Río San Pedro s/n., 11510Puerto Real, Cádiz, Spain; ‡ Computation-based Science and Technology Research Center, The Cyprus Institute, 20 Constantinou Kavafi Str., 2121Nicosia, Cyprus; § Institute of Applied and Computational Mathematics, Foundation for Research and Technology Hellas, GR-70013Heraklion, Crete, Greece; ∥ Department of Mathematics and Applied Mathematics, University of Crete, GR-71409Heraklion, Crete, Greece

**Keywords:** star polymers, penetrability, impenetrable region, atomistic simulations, colloidal-like character

## Abstract

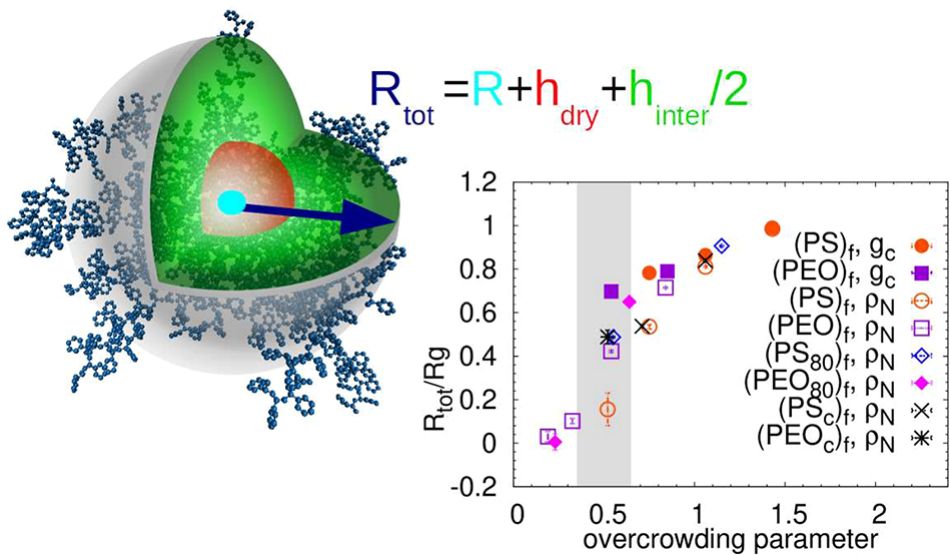

The importance of microscopic details in the description of the behavior of polymeric nanostructured systems, such as hairy nanoparticles, has been lately discussed via experimental and theoretical approaches. Here we focus on star polymers, which represent well-defined soft nano-objects. By means of atomistic molecular dynamics simulations, we provide a quantitative study about the effect of chemistry on the penetrability of star polymers in a melt, which cannot be considered by generic coarse-grained models. The “effective softness” estimated for two dissimilar polymers is confronted with available literature data. A consistent picture about the star penetrability can be drawn when the star internal packing is taken into consideration besides the number and the length of the star arms. These findings, together with the recently introduced two-layer model, represent a step forward in providing a fundamental understanding of the soft character of stars and guiding their design toward advanced applications, such as in all-polymer nanocomposites.

Regular star polymers have been used for decades as model structures for more complex architectures, since they represent well-defined objects composed of *f* equally long chemically identical arms.^1−4^ It is generally accepted that star-like polymers with a low functionality, *f*, bear some resemblance to linear polymer chains, while multiarm stars exhibit colloidal-like character due to their low degree of penetrability.^5−7^ The broad spectrum of properties in stars, being between these two limiting cases, is generally referred to as the “linear-to-colloidal transition”. This transition correlates with both star functionality and arm length.^8,9^ In addition, the character of the central branch point^10,11^ determines the internal structure and consequently the properties of star-shaped molecules.

The experimental investigation of the internal star morphology is nontrivial, and one has to rely on computational and theoretical models when interpreting results; the latter ones typically involve several approximations.^2,12−14^ A melt state is particularly laborious to study because many-body interactions are usually not accounted for in single-molecule models. Also, elaborate experimental practices, such as scattering techniques, are necessary to get basic characteristics like the star radius of gyration *R*_g_.^15,16^

Recently, a two-layer model has been presented for grafted nanoparticles in a melt, which divides the space around nanoparticles into an impenetrable core and an interpenetration layer,^17^ similarly to the work on hyperstar polymers.^18^ Both grafted nanoparticles and hyperstar polymers, resemble polymer brushes, in which the chains have one end anchored to a substrate or a polymer backbone and, due to their high grafting density, tend to pack closely, forming “dry” impenetrable regions of a given brush thickness.^19,20^ The brush thickness (or height) was shown to be a function of an overcrowding parameter *x*, which combines various structural characteristics.^17,21^

Simulation techniques have been used extensively in the past to investigate the linear-to-colloidal transition in star melts, usually via generic coarse-grained models.^9,22−25^ In the pioneering work of Pakula and co-workers^9,22,23^ the functionality was discussed as the main parameter affecting the star dynamical behavior, and an impenetrable central region was considered significant for stars with *f* > 24. In refs (24 and 25), the authors observed a configurational transition from a chain-like anisotropic to a more spherically symmetric structure for stars with *f* ≥ 8. The above generic models are expected to describe accurately properties at length scales of about, and above, the Kuhn length *l*_k_ (a few monomers), and consequently they are not able to capture fine details in local packing at the monomeric scale characteristic for specific polymer types. Moreover, they typically describe in a rather crude way the structure of the central branch point of the star.

Atomistic models can overcome the above limitations of the generic models as they fully capture the chemical specificity (type of monomers). Recently, atomistic simulations of polystyrene (PS) and poly(ethylene oxide) (PEO) stars in a melt demonstrated that the geometric constraints stemming from the star-like architecture affect the conformational properties of PS stars more than PEO stars with the same functionality due to the presence of the bulky styrene monomer in the former systems.^26^

The above observations lead to specific open questions related to the soft character of star polymers in a melt, which have not been addressed so far by the generic models, such as, for example: How does the star penetrability depend on the chemistry-specific details? Is the linear-to-colloidal transition in star polymer melts universal or does it depend on the underlying chemistry?

Here we address the above questions via large-scale atomistic molecular dynamics simulations. We avoid discussing well-documented theoretical and generic models of stars in solutions summarized, e.g., in refs (2 and 5), and we rather focus on a chemistry-specific description of multiarm stars in a melt. We simulate homopolymer melts composed of unentangled regular stars. Two polymer types, PEO and atactic PS, were chosen because of their distinct monomeric structure. The data set consists of the main data set (DS1) of stars with 40 monomers per arm (*N*_arm_ = 40) and *f* = 8, 16, 32, labeled as (PS)_f_ and (PEO)_f_, and (DS2) additional test samples, which differ from the DS1 (a) by the number of stars in the system and *f* (*f* = 4), (b) by the arm length (*N*_arm_ = 80), and (c) by the radius *R* of the central dendritic kernel. The system characteristics are listed in Table S1 in the Supporting Information. To provide accurate quantitative predictions about the star spatial arrangement, we simulate melts consisting of hundreds of stars. They are built from smaller systems, whose preparation is illustrated in Figure 1a–c and described in detail elsewhere.^26^ The preparation of the large systems studied here (Figure 1d) is described in the Supporting Information.

**Figure 1:**
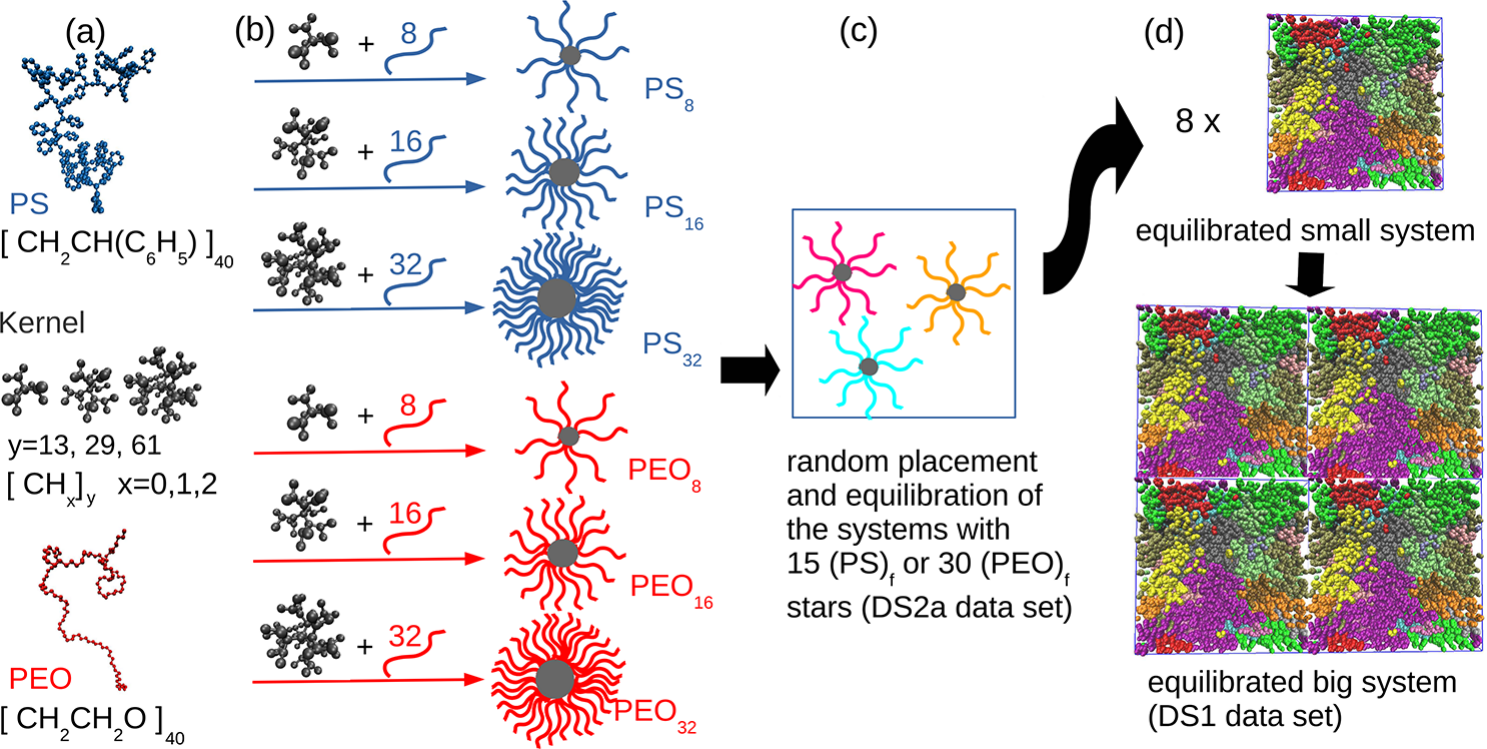
Schematic representation of the preparation of model star polymer melts. Steps (a–c) refer to the generation and equilibration procedure;^26^ systems presented here were prepared by step (d). (a) Snapshots and chemical formulas of the building units, i.e., central carbon kernels, a PS arm, a PEO arm. (b) Preparation of the systems and their notation. Note that a different dendritic kernel is used for stars with different *f*. (c) Preparation of the systems with 15 or 30 molecules described in ref (26). (d) Generation and equilibration of the large-scale star polymer melts containing up to 240 stars. The snapshots were created by VMD.^27^

First, we investigate the intramolecular star structure through the single-star form factor *W*(*q*) calculated as
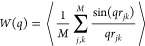
1where *q* is a wave vector, *r*_*jk*_ is the distance between the atoms *j* and *k*, *M* is the number of atoms per star, and the brackets denote an average over all molecules and time frames. The form factor probes the internal packing at different characteristic length scales represented by 1/*q*. At distances much bigger than the particle size *R*_g_ (1/*q* ≫ *R*_g_) *W*(*q*) gives the information about the number of scattering objects *M* in the sample (here the number of atoms), i.e., *W*(*q*)/*M* → 1 for *q* → 0. In Figure 2a *W*(*q*)/*M* exhibits a two-step decay for *R*_g_ ≥ 1/*q* ≥ *l*, where *l* is the average bond length, which agrees qualitatively with previously published experimental and theoretical works on stars under different solvent conditions^2,5,10^ and in a melt.^22^ At length scales larger than the monomeric size, a fractal regime *W*(*q*) ≈ *q*^–1/ν^ independent of the functionality is expected for stars in solution. In the fractal regime the average number of particles within a sphere of radius *R*_w_ = 1/*q* varies as *R*_w_ to some fixed power *D*, which denotes the fractal dimension.^28^ A Gaussian chain, for which the intermonomer distances obey a Gaussian distribution function, has a fractal dimension *D* = 2 and ν = 0.5. For more compact objects, such as crumpled globules,^29^*D* = 3 and ν = 1/3, i.e., *W*(*q*) ≈ *q*^–1/0.33^. For the star melt systems shown in Figure 2 such a fractal regime with the exponent around 0.5 can be identified for (PEO)_f_ in a *q* range between 2 and 7 nm^–1^, while for (PS)_f_ the *W*(*q*) shows such a tendency only in a very narrow *q* range around *q* ≈ 2 nm^–1^. The PS arms of approximately seven Kuhn segments^30^ are apparently insufficiently long to follow the asymptotic (Gaussian-like) behavior. A sharp decay, typical for compact molecules, is followed for larger values of *q*. As the decay is observed at 1/*q* ≈ *l*_k_ (*l*_k_(PS) = 1.4 nm^30^) we hypothesize that it is related to the bulky character of the PS monomers. Concerning the intensity drop at intermediate length scales, we decomposed *W*(*q*) into the scattering contributions coming from the same-arm, *W*_intra_, and interarm, *W*_inter_, correlations in Figure 2b. The *W*_intra_ would be an analogy of the experimental scattering function obtained for stars in a melt with labeled arms.^15^ Here, *W*_intra_ intensities are identical for all stars of the same polymer type within the obtained accuracy. The decay of *W*_inter_ is more abrupt for (PS)_f_ than for (PEO)_f_, which agrees with a tighter packing of PS arms reported recently.^26^ The shape of *W*_inter_ for stars with *f* = 8 is identical with the interchain scattering of linear chains of the same chemistry (data not shown), confirming that the packing of the arms in these stars is similar to the linear chains in bulk. As *f* increases, a steeper drop of *W*_inter_ is observed, which indicates an increasing impact of geometrical constraints imposed by the star-like architecture.

**Figure 2:**
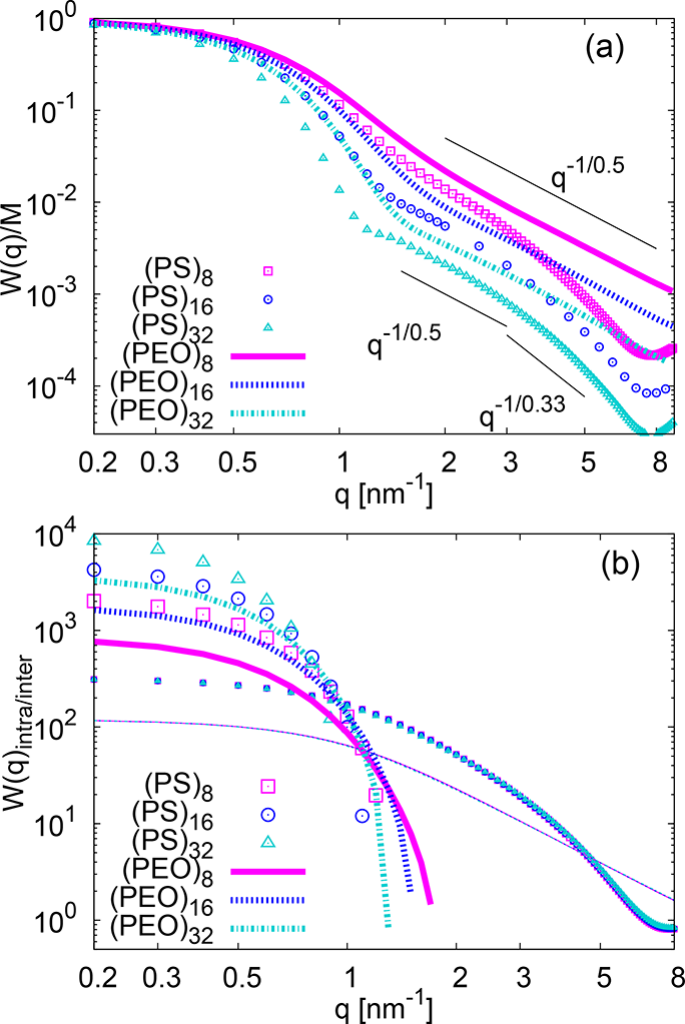
Single-star form factor in (PEO)_f_ and (PS)_f_ star melts. (a) Total form factor including correlations between all atoms *M* in a star. The solid black lines are guides to the eye, and they represent fractal regimes expected for a chain with Gaussian statistics (ν = 0.5) and for a crumpled globule (ν = 1/3). (b) Form factor divided into the scattering contributions of the intraarm *W*_intra_ (small points, thin lines) and interarm *W*_inter_ (large points, thick lines) spatial correlations. Note that the data for *W*_intra_ for different systems overlie.

The most intuitive way of quantifying the degree of interpenetration of two objects is to compute the number of their contacts. We counted for each monomer the number of neighboring monomers coming from the same, *n*_intra_, or from the surrounding, *n*_inter_, stars in a sphere of radius equal to the *l*_k_ (*l*_k_(PEO) = 0.98 nm and *l*_k_(PEO) = 1.4 nm^30^). Then, we classified the monomers into three groups: *n*_intra_/*n*_inter_ < 1, *n*_intra_/*n*_inter_ > 1, and *n*_inter_ = 0. The visual inspection of the last two populations in Figure 3 leads to some preliminary conclusions: (a) there is no “dry” region with *n*_inter_ = 0 in the stars with *f* = 8; (b) for the stars with *f* = 32 the monomers with intramolecular contacts dominate (compare the colored with the blank regions in the right snapshots in Figure 3). The results shown in Figure 3 agree qualitatively with the simulation work on miscibility of identical stars at high volume fractions,^31^ in which the number of contacts with surrounding stars decreased with increasing *f*.

**Figure 3:**
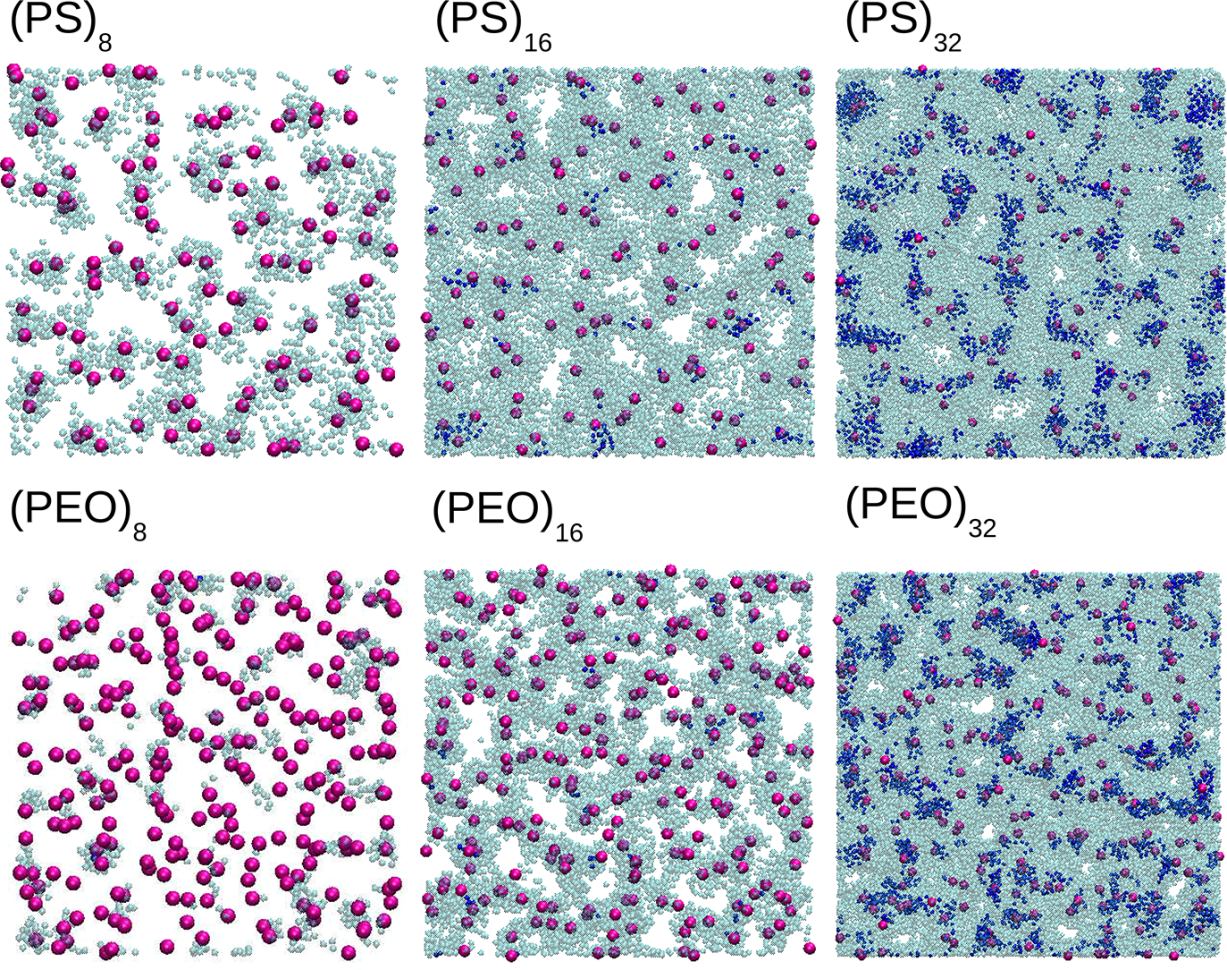
Ratio *n*_intra_/*n*_inter_ projected onto one randomly selected snapshot per simulated system. The magenta beads correspond to the central carbon of the dendritic kernel, the cyan transparent beads represent the monomers for which the ratio *n*_intra_/*n*_inter_ is bigger than 1. The monomers for which *n*_intra_/*n*_inter_ < 1 are omitted for clarity. For the blue monomers *n*_inter_ = 0. *n*_intra_ denotes the number of neighboring monomers belonging to the arms of the same star in a sphere of radius *l*_k_, and *n*_inter_ indicates the number of neighboring monomers belonging to the arms of penetrating stars. The size of the beads does not correspond to the size of the actual monomers. The snapshots were created by VMD.^27^

Polymer-grafted nanoparticles resemble star-like molecules when the size of the central particle is small in comparison to the length of the grafted chain. Following such an analogy, we quantify the brush thickness via monomer density profiles and radial distribution functions of star centers, as proposed by the two-layer model of hairy nanoparticles.^17^ In that work, the overall radius *R*_tot_ of a two-layer object is a sum of the radius of the impenetrable layer, *h*_dry_, the radius *R*, and the half of the interpenetrable region, *h*_inter_.
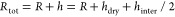
2Furthermore, in ref (17) the intersection point of the intramolecular ρ_intra_(*d*) and the intermolecular ρ_inter_(*d*) components of the number monomer density profiles was used for the brush height (*h*) estimation. In Figure 4a,b the intersection point of ρ_intra_(*d*) and ρ_inter_(*d*) is placed at *d* = *R* + *h* = *R*_tot_, because our profiles are plotted with respect to the distance from the central carbon of the dendritic kernel, which is, in principle, penetrable. In addition to the two-layer model, we briefly discuss in the Supporting Information the predictions of the standard blob model (Figure S1) and the method for *h* estimation proposed in ref (22) (Figure S2). The percentage of the monomers packed in a sphere with the given brush radius is shown in Figure S3 in the Supporting Information. The results confirm a higher penetrability of PEO stars in comparison to the PS ones and a very high compactness of (PS)_32_.

**Figure 4:**
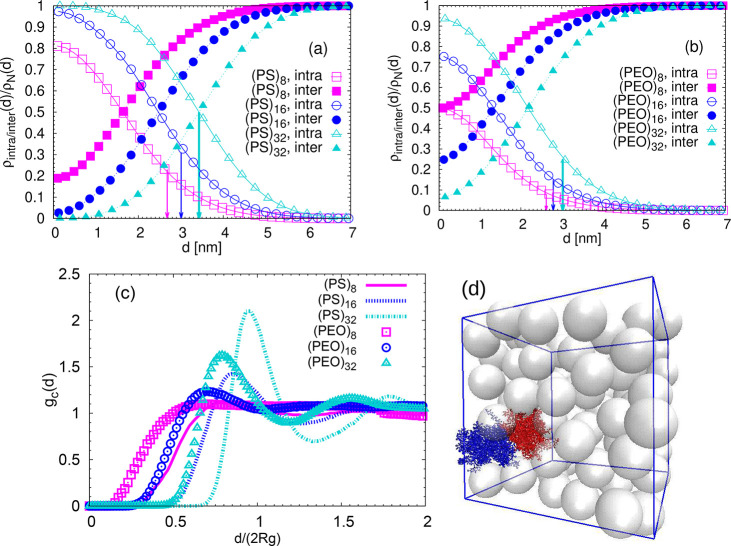
(a, b) Inter- and intramolecular number monomer density profiles of star polymer melts. The data are normalized by the same quantity, which includes both components, ρ_N_(*d*) = ρ_intra_(*d*) + ρ_inter_(*d*), for a better comparison of different polymer types. The distance *d* corresponds to the radial distance from the central carbon unit in the star. The arrows indicate the radius of gyration of the stars in the systems with the same color code. (c) Radial distribution functions of the distances between the central carbons of stars in a melt. (d) A representative snapshot of two (PS)_32_ stars inside of the simulation box. The surrounding stars are represented as transparent spheres with radius equal to the average radius of gyration of (PS)_32_ stars. The snapshot was created by VMD.^27^

Besides the internal packing, the soft character of an object is reflected in its spatial arrangement with respect to its neighbors. To probe such an arrangement we plot the pair radial distribution function of the central carbon atoms of the stars, *g*_c_(*d*), in Figure 4c. Analogous results are obtained if, alternatively, the center-of-mass of the star is used as a central point, alike to some simulation studies.^22,32^ The first maximum in Figure 4c represents the most likely packing arrangement of two stars, and its position, *d*_max_, can be associated with the brush height as *d*_max_/(2*R*_g_) = (*h* + *R*)/*R*_g_ = *R*_tot_/*R*_g_. By comparing the positions of the first maxima (i.e., the quantity (*h* + *R*)/*R*_g_), we conclude that, as to the brush extension, the (PEO)_f_ stars are more penetrable than their PS analogues with the same *f*. The same qualitative conclusion can be reached when comparing the intersection points in Figure 4a,b to *R*_g_. Additionally, the maximum for the (PS)_8_ star is barely detectable, in contrast to Figure 4a, where the intersection was found at *d* > *R* (see Table S1 for the values of *R*). Note that, in ref (22), the maximum was clearly detected for stars with *f* > 8 and a vague peak was visible for *f* = 8.

With increasing functionality multiple shallow minima appear in *g*_c_(*d*), indicating local arrangements. The typical star–star distance for the most compact (PS)_32_ is around 2*R*_g_; i.e., if these stars were coarse-grained into spherical objects with the radius of *R*_g_, they would be in close contact in a melt. This situation is visualized in Figure 4d. Note that such information is needed when estimating an effective potential between highly coarse-grained stars.^5,7^ Also, the visual inspection of the snapshot allows us to verify the random distribution of the molecules in the box. This observation was quantified for all systems in Figure S4 of the Supporting Information.

Results from intra- and intermolecular analysis reveal different quantitative behavior of stars with constant *f* and *N*_arm_ made of distinct polymer types. Therefore, to combine together different structural characteristics we define an overcrowding parameter as
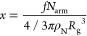
3where . The parameter *x* represents the ratio of the number of monomers in a single star (i.e., *fN*_arm_) over the number of monomers that would occupy the same volume in an unperturbed melt. The overcrowding parameter *x* defined via eq 3 is in line with a physical interpretation of the same parameter obtained from the analytical solution of the two-layer theory (see eq S5, Figure S5, and the related discussion in the Supporting Information).

The *R*_tot_ obtained from DS1, DS2(a,b,c), and from the published data are plotted in Figure 5a against *R*_g_. It is computationally demanding to obtain reliable results from the *g*_c_ analysis; therefore, we only show data for systems with an easily detectable maximum and good statistics. This plot quantitatively confirms the observations in Figure 4. Comparing the two methods of estimation, the values of *R*_tot_ obtained from the density profiles lie systematically below the values obtained from *g*_c_. For hairy nanoparticles,^17^ the two methods gave identical results, which might be due to their more compact character and a different functional form of ρ_intra_(*d*) in comparison to stars. Note also that the two-layer model assumes a spherical brush, (PS)_f_ are more spherical than (PEO)_f_, and the asphericity decreases with *f*.^26^ To eliminate the arm length dependency visible in Figure 5a, we present the ratio *R*_tot_/*R*_g_ as a function of *x* in Figure 5b. In this representation the data divide into two regions of low and high *R*_tot_/*R*_g_ ratios, with the turning point near *x* ≈ 0.5. Due to the limited range of *f* and *N*_arm_ studied here, we avoid drawing conclusions about a specific combination of parameters marking the transition point. Nevertheless, much more consistent results are seen when judging the soft character of stars by their *x* parameter, which reflects their local packing better than *f*. Comparing stars with the same *f* and a similar number of Kuhn segments per arm (e.g., (PEO)_8_ and  or (PEO)_32_ and  in Figure S6 in the Supporting Information), the discrepancies in *R*_tot_/*R*_g_ are bigger than when stars with similar *x* are compared (e.g., the purple open square for (PEO)_16_ and the blue open diamond for  for *x* ≈ 0.55 in Figure 4b). Concerning the experimental evidence about the linear-to-colloidal transition, it has been mostly judged by the values of *f*. Interestingly, the linear rheology data on PEO stars could be well-described by the Milner–McLeish model with no interarm interactions for *f* up to 32,^33^ while the rheological spectra of PS stars with the same range of *f* but slightly shorter arms reported an appearance of colloidal-like behavior.^8^ Since the stars in refs (8 and 33) contain the carbosilane dendrimers^1^ analogous to our dendritic kernels, we take the size of our kernels as the lowest estimation of the *R* and calculate *x* from eq S4 in the Supporting Information^21^ by using two sets of parameters (*l*_k_, ν_0_), published in refs (30 and 34). This rough estimation gives us the higher boundary of *x* (*R* is bigger in experimental samples due to the longer Si–C than C–C bond used here).^35^ All PEO stars from ref (33) have *x* < 0.5 independently of the chosen set of parameters. The so-called transitional dynamics observed for PS stars in ref (8) correspond to 0.3 ≤ *x* ≤ 0.7. These estimations provide more general trends than exact numbers; however, they give us some hint about the values of *x* for experimental samples very alike to those simulated here. The data extracted from ref (22) should be also taken with a pinch of salt, as due to the limited number of points in those density profiles our estimation of the intersection points is loaded with a considerable error. Nevertheless, we observe remarkably coherent tendencies for our atomistic data, the published generic models, and the two experimental studies.

**Figure 5:**
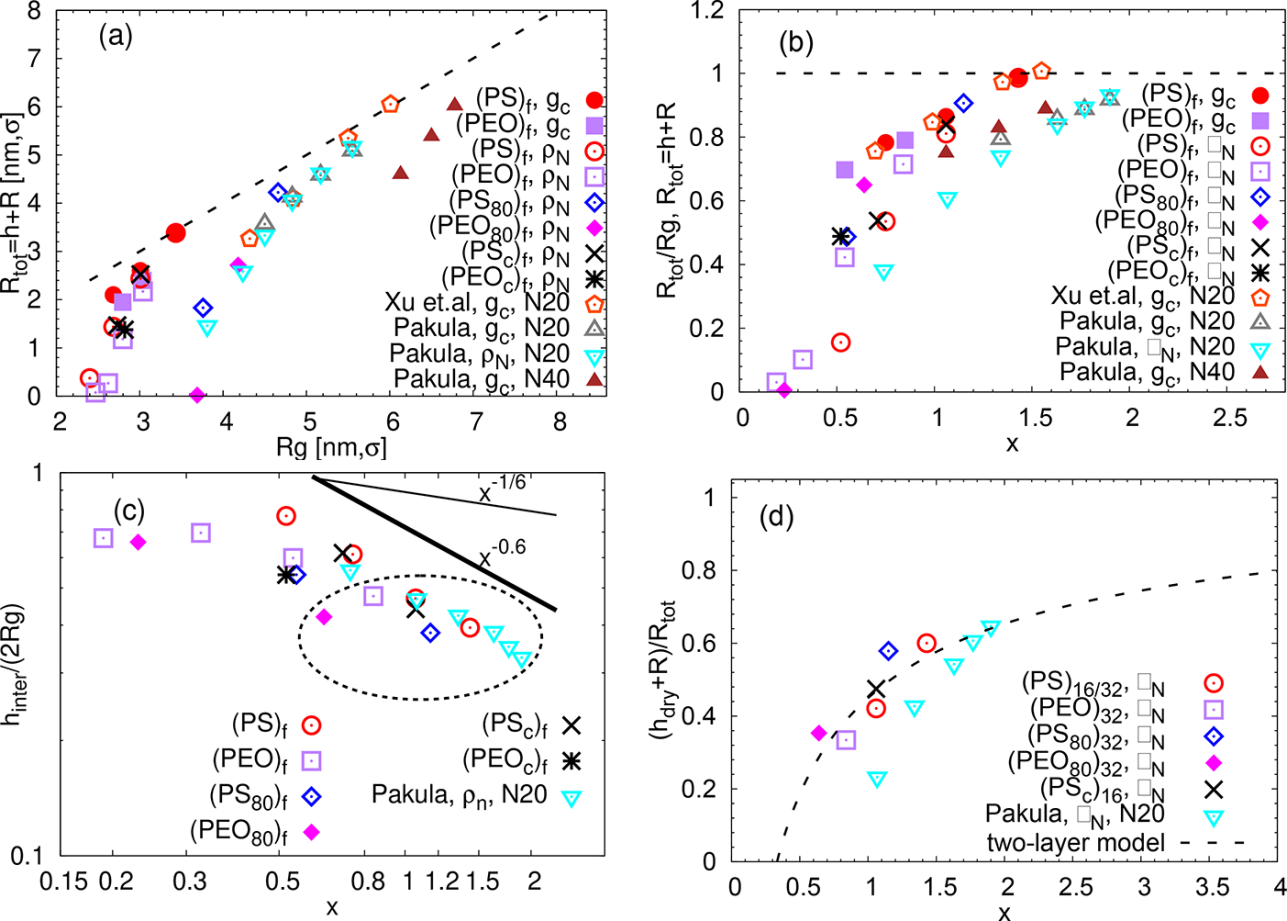
(a) The total size of the two-layer object *R*_tot_ defined as a sum of the brush height *h* and the size of the central particle *R*, with respect to the radius of gyration *R*_g_ of the systems. The dashed line indicates the relation *R*_g_ = *R*_tot_. (b) Ratio *R*_tot_/*R*_g_ as a function of the overcrowding parameter *x*. (c) The thickness of the interpenetrable region *h*_inter_/2 normalized by the radius of gyration *R*_g_ as a function of *x*. For the points highlighted by the ellipse *h*_inter_/2 < *R*_tot_. The thick and the thin line are guides to the eye. (d) The sum of the dry brush height *h*_dry_ and the size of the central particle *R* normalized by *R*_tot_ and plotted as a function of *x*. The labels in the legend refer to refs (32) and (22) and the number of coarse-grained units per arm (*N*). The method for obtaining the brush height from these publications is also indicated. The error bars related to the systems simulated here are of the order of the point size.

We decompose the brush height *h* obtained from the density profiles into *h*_dry_ and *h*_inter_/2 as described in ref (17) and in Figure S7a in the Supporting Information. The so-obtained *h*_inter_/2 values are plotted as a function of *x* in Figure S7b. They follow closely the *h*_inter_/2 ≈ *l*_k_*N*_k_^1/2^*x*^–1/6^ prediction of Kapnistos,^18^ with a scaling prefactor seemingly depending on various star characteristics. Since for the highly penetrable stars *h*_inter_/2 > *R*_tot_ (see below), we use *R*_g_ as a scaling factor in Figure 5c. As seen in this representation, the interpenetrable region in the penetrable stars with low *x* occupies a considerable part of the molecule. More specifically, we were able to estimate *h*_dry_ from the expression *h*_dry_ + *R* = *R*_tot_ – *h*_inter_/2 only in six systems with *x* > 0.5 (highlighted in Figure 5c). Their normalized values of (*h*_dry_ + *R*)/*R*_tot_ are plotted in Figure 5d and in Figure S6b together with the theoretical prediction. The results for our systems of compact stars are in remarkable agreement with the two-layer model prediction. Taking into consideration the errors in the extraction of the intersection points from ref (22), the two-layer model also provides a reasonable description for this generic model.

In summary, our results emphasize the importance of chemical specificity on the properties of star polymer melts and particularly in the linear-to-colloidal transition. We showed that the soft character of a variety of stars of different *f*, *N*_arm_ and polymer types depends on the intra- and intermolecular packing in the system. Without having this detailed information, the star can be classified as penetrable according to the value of the overcrowding parameter, which was also introduced by the recent two-layer theory applied to hairy nanoparticles in a melt. This classification and the verification of the applicability of the two-layer theory contributes significantly to the accurate estimation of the degree of softness in star melts, which is a key parameter in emerging applications of such materials, as, for example, in nanomedicine,^36^ separation membranes,^37^ and in all-polymer nanocomposites.^38^

## Supporting Information Available

Additional figures together with the description of the systems and the equilibration method (PDF)
