# Supplementary material for: Soft Character of Star-Like Polymer Melts: From Linear-Like Chains to Impenetrable Nanoparticles
Source: Nano Lett. 2023 Jan 9;23(4):1608–14. doi: 10.1021/acs.nanolett.2c04213 (PMC9951242; doi:10.1021/acs.nanolett.2c04213)
Supplement: Supplementary file 1 — nl2c04213_si_001.pdf [file nl2c04213_si_001.pdf]

SUPPLEMENTARY INFORMATION: Soft character of star-like polymer melts: From linear-like chains to impenetrable nanoparticles

Petra Bačová,[1,2,3,*] Eirini Gkolfi,[2,3,4] and Vagelis Harmandaris[2,3,4,†]

[1] *Departamento de Ciencia de los Materiales e Ingeniería Metalúrgica y Química Inorgánica, Facultad de Ciencias, IMEYMAT, Campus Universitario Río San Pedro s/n., Puerto Real, Cádiz 11510, Spain*

[2] *Computation-based Science and Technology Research Center, The Cyprus Institute, 20 Constantinou Kavafi Str., Nicosia 2121, Cyprus*

[3] *Institute of Applied and Computational Mathematics (IACM), Foundation for Research and Technology Hellas (FORTH), GR-70013 Heraklion, Crete, Greece*

[4] *Department of Mathematics and Applied Mathematics, University of Crete, GR-71409 Heraklion, Crete, Greece*

[*] petra.bacova@uca.es

[†] v.harmandaris@cyi.ac.cy

I. PREPARATION AND EQUILIBRATION OF THE MODEL STAR POLYMERS IN MELT

The preparation of the small model PS and PEO star systems in melt, which serve as building units for the systems studied here, was described in detail elsewhere. [1] Here we only briefly describe the methodology depicted in Fig.1(a,b,c) of the main manuscript. The star-shaped topology was prepared by attaching fully stretched chains of 40 monomers consisting of either styrene or ethylene oxide monomers into a central dendritic structure. The central dendritic structure, also called kernel, is built of C, CH and CH_2 units and it is made of 1 to 3 generations depending on the functionality (number of arms, f) of the prepared star. Overlaps and local tension, that can occur by forming such a topology, were eliminated by a serie of short runs consisting of energy minimization and molecular dynamics with a low time step of 0.1 fs. Then, the stars were randomly placed in a simulation box. The equilibration was performed by combining NPT runs and runs with stochastic dynamics at higher temperatures, as described in detail in ref. [1]. The production runs of 100 ns were performed at the temperature of 600K for $(\mathrm{PS})_f$ and 450K for $(\mathrm{PEO})_f$, employing Nosé-Hoover thermostat and Parrinello-Rahman barostat at a constant pressure of 1 atm. The same procedure was used for the DS2a systems and for the systems consisting of 15 and 30 stars in DS2c data set (see Tab. S1 below).

The last configurations from the production runs of the systems described above and in ref. [1] were taken and used as initial configurations for the preparation of the bigger systems (i.e., for the DS1, DS2b and $(\mathrm{PS}_c)_8$ listed in Tab. S1). The coordinates of the initial configuration were unwrapped to retain the information about the connectivity. We replicated this initial configuration 8 times to create a new, bigger cubic simulation box as depicted in Fig.1(d) of the main manuscript. The number of stars in so-prepared system is listed in Tab. S1. In order to avoid overlaps of unwrapped molecules, an extra gap was kept between the replicated small boxes. To eliminate this gap and to obtain homogeneous systems, we run an equilibration run in the NPT ensemble. The NPT simulation of PEO systems lasted 100 ns at 550 K with the pressure equal to 1 atm. The Langevin dynamics was used for the NPT run of PS systems for 50 ns, with the temperature of 700K and the pressure of 1 atm to speed up the equilibration. The box size was adjusted within the first 4 nanoseconds and then the box size fluctuated around an average value, indicating a successful elimination of the gaps. In the end of these equilibration runs, the density profiles along the sides of the simulation box were calculated to confirm the homogenous character of the systems. Before the production run, we set a pre-production run of 100 ns with the same settings as the production run. The production runs were 500 ns long and were performed at 600 K in the case of PS systems and at 450 K in the case of PEO stars. The temperature was maintained by Nosé-Hoover thermostat and the pressure was kept at 1 atm by Parrinello-Rahman barostat. All the simulations were performed by Gromacs simulation package [2] with a timestep of 1 fs.

II. COMPARISON TO THE THEORETICAL SCALING PREDICTIONS

A. Comparison to the standard blob model

The standard blob model of an isolated regular homopolymer star applies geometrical arguments to predict three regions of different scaling of the density (concentration) profile, as a function of distance from the center of a star molecule. [3–5] The framework was developed for stars in solutions with ideal arms following random walk statistics and therefore, it is not expected to provide quantitative predictions for star melts studied here. However, as it has been used previously for the interpretation of the experimental data in star melts [6], we report our data together with the scaling predictions in Fig. S1.

B. Comparison to the Gaussian approximation

In Fig. S2 we fit the $\rho_{\mathrm{intra}}(d)$ data to the Gaussian function, similarly to ref. [7] where this approach was used to estimate the extension of the impenetrable region. Our profiles exhibit a Gaussian shape only in a certain range of d, therefore, we do not further discuss this method here.

data set	system	f	R [nm]	ρ_N [nm^{-3}]	N_s	$N_{\rm arm}$	x (eq. S5)	Rg [nm]	R_{tot} (g_c)[nm]	R_{tot} (ρ_N) [nm]	$h_{\rm inter}$ [nm]
DS1	$(PS)_8$	8	0.4735	5.27	120	40	0.75	2.683± 0.004	2.1±0.07	1.44±0.02	1.64±0.02
	$(PS)_{16}$	16	0.5755	5.27	120	40	1.07	3.008± 0.002	2.6±0.06	2.44±0.01	1.41±0.01
	$(PS)_{32}$	32	0.7085	5.27	120	40	1.43	3.433± 0.003	3.4±0.14	3.384±0.001	1.353±0.002
	$(PEO)_8$	8	0.4735	13.02	240	40	0.33	2.621± 0.004	-	0.27±0.03	1.83±0.01
	$(PEO)_{16}$	16	0.5755	13.02	240	40	0.54	2.797± 0.003	1.95±0.003	1.18±0.01	1.68±0.01
	$(PEO)_{32}$	32	0.7085	13.02	240	40	0.84	3.038± 0.003	2.4±0.04	2.172±0.005	1.446±0.003
DS2a	$(PS)_4$	4	0.3526	5.27	15	40	0.52	2.4± 0.003	-	0.37±0.18	1.85±0.11
	$(PEO)_4$	4	0.3526	13.02	30	40	0.19	2.47± 0.015	-	0.07±0.06	1.67±0.04
DS2b	$(PS_{80})_8$	8	0.4735	5.27	120	80	0.55	3.76±0.01	-	1.83±0.01	2.04±0.01
	$(PS_{80})_{32}$	32	0.7085	5.27	120	80	1.15	4.66±0.01	-	4.225±0.006	1.780±0.003
	$(PEO_{80})_8$	8	0.4735	13.02	240	80	0.23	3.69±0.01	-	0.02±0.14	2.43±0.08
	$(PEO_{80})_{32}$	32	0.7085	13.02	240	80	0.64	4.18±0.03	-	2.72±0.01	1.76±0.01
DS2c	$(PS_c)_8$	8	0.7085	5.27	120	40	0.71	2.73±0.01	-	1.47±0.01	1.68±0.01
	$(PS_c)_{16}$	16	0.7085	5.27	15	40	1.06	3.01±0.01	-	2.52±0.01	1.33±0.02
	$(PEO_c)_{16}$	16	0.7085	13.02	30	40	0.52	2.82±0.02	-	1.38±0.08	1.53±0.08

TABLE S1. Details of the main data set DS1 and the additional data sets, DS2a, DS2b and DS2c. f stands for the functionality of each star, R for the radius of the kernel, ρ_N for the number monomer density, N_s for the number of stars in the simulation box, $N_{\rm arm}$ for the number of monomers per arm, x for the overcrowding parameter and Rg for the average radius of gyration of the molecule. The total size of the two-layer object R_{tot} estimated by the g_c method and the ρ_N method is also listed, together with the width of the interpenetrable layer $h_{\rm inter}$. The error bars were calculated by a block averaging method from 4 blocks.

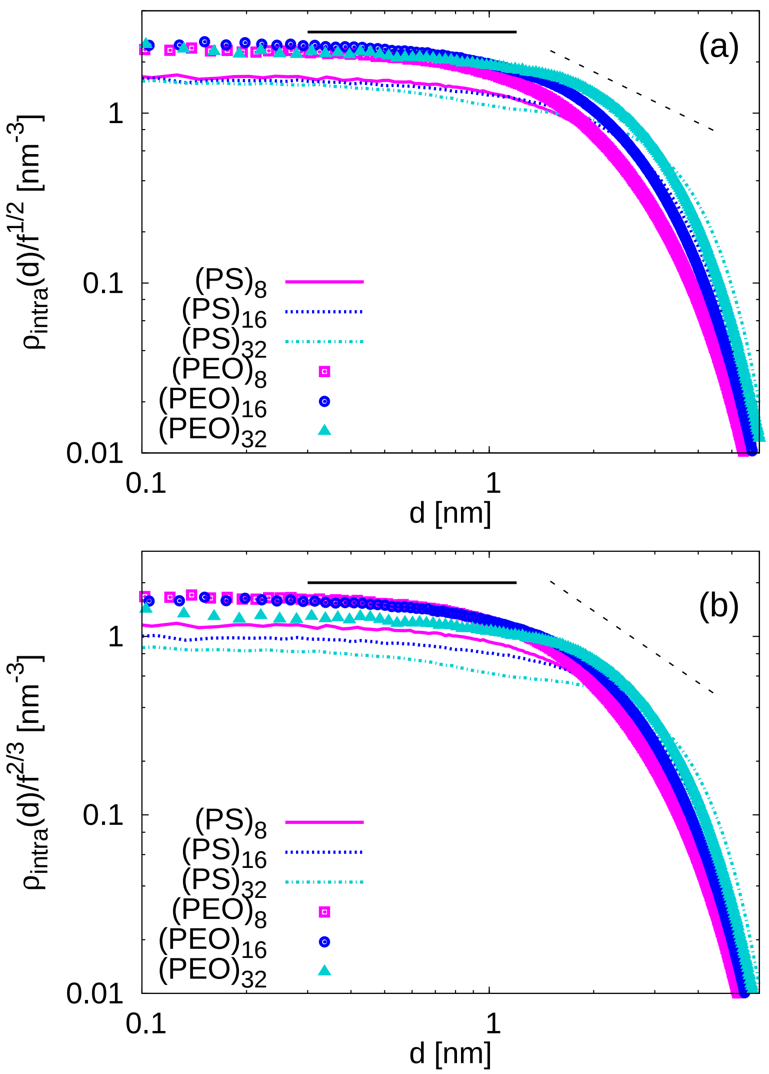

FIG. S1. Intramolecular number monomer density profiles normalized by (a) $f^{1/2}$ and (b) $f^{2/3}$ as a function of distance d from the central star carbon. The horizontal solid line in both graphs indicates the independence of the profile on d, which is expected for the impenetrable (core) region. The dashed line illustrates the scaling (a) $\rho_{\rm intra} \sim d^{-1}$ typical for the unswollen region and (b) $\rho_{\rm intra} \sim d^{-4/3}$ expected for the swollen region.

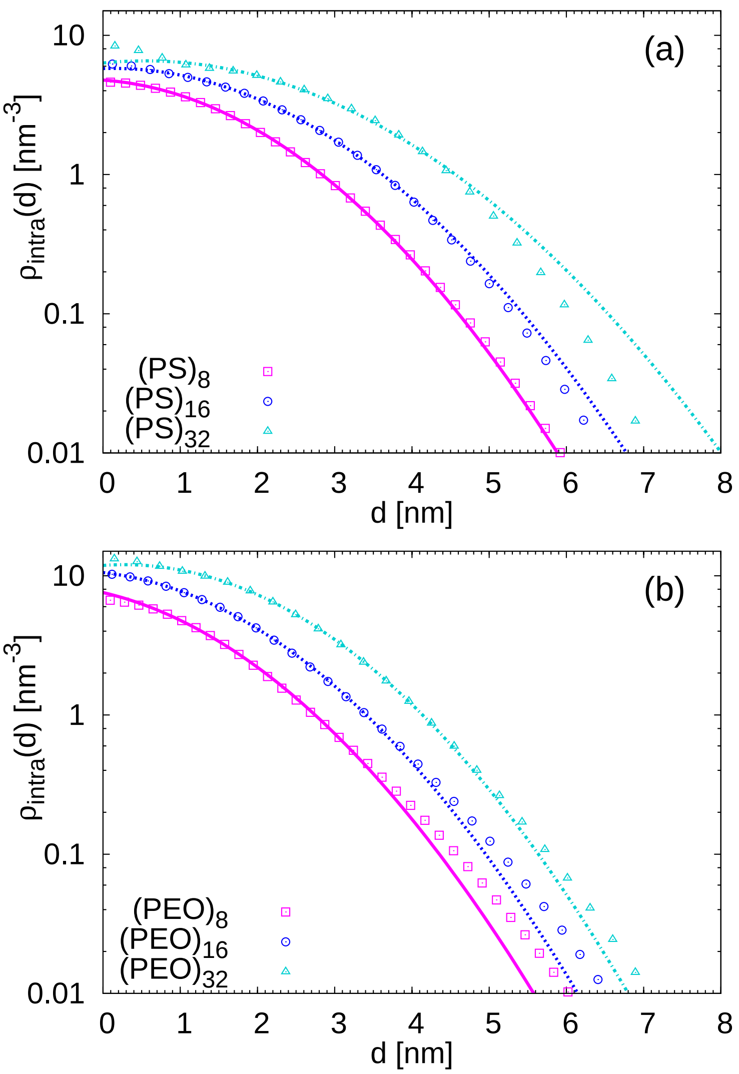

FIG. S2. Intramolecular number monomer density profiles as a function of distance d from the central star carbon for (a) $(\mathrm{PS})_f$ and (b) $(\mathrm{PEO})_f$ stars. The lines in both graphs represent Gaussian fits of the profiles in the range of $d \geq R$. Similar procedure was followed in ref. [7], where the position of the Gaussian maximum d_{Gauss} was deemed to represent the range of the impenetrable core, h_{dry}. However, such a quantitative estimation was avoided in this work because of the following reason: the range of d where the profiles can be described by a Gaussian decay varies for both chemistries, particularly at large d. Therefore, as the position of the maximum strongly depends on the selected range of d for the fitting procedure, the estimated h_{dry} has a huge error and its value is unreliable. Note also, that the density profile in ref. [7] is measured from the center of mass of the molecule, while here we use the central carbon atom.

III. ADDITIONAL DATA RELATED TO THE INTRAMOLECULAR ANALYSIS OF STAR-SHAPED MELTS

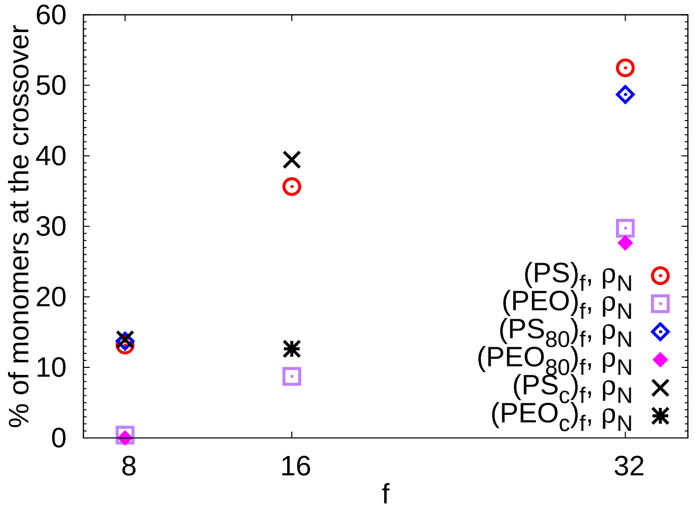

FIG. S3. Percentage of the monomers contributing to the intramolecular density profile before the intersection point as a function of the functionality, f. Note that the limit of 100% of the monomers would correspond to a sphere with a so-called corona radius R_c [8], which is of the order of the longest arm end-to-end vector in the system and it is more than factor of 2 larger than the average Rg (see Tab. S1).

IV. ADDITIONAL DATA RELATED TO THE INTERMOLECULAR ANALYSIS OF STAR-SHAPED MELTS

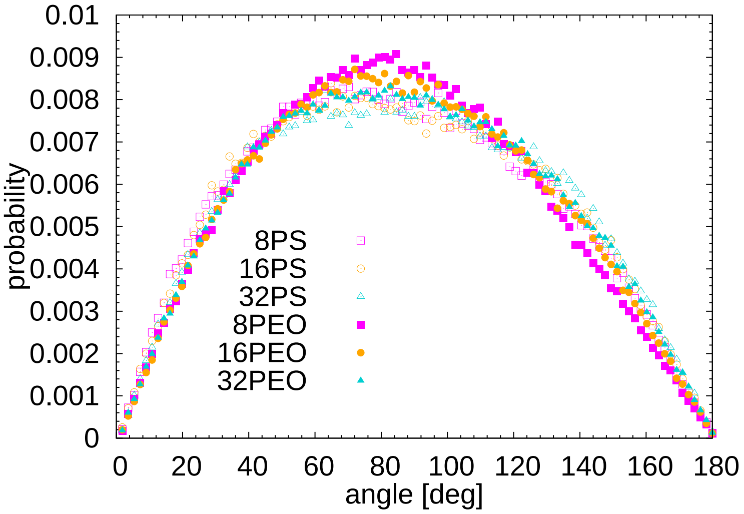

FIG. S4. Probability distribution function of the angle between the main axes of the nearest neighbor molecules. The biggest eigenvector of the radius of gyration tensor was chosen as the main axis of the molecule. Only a negligible tendency for a preferential alignment for $(PEO)_8$ stars, which are the least spherical ones among the studied systems [1], is observed.

V. OVERVIEW OF THE TWO-LAYER MODEL

The two-layer model assumes a spherical shape of the polymer layer around the central particle of radius R [9]. The total volume of the particle is expressed as:

$$\frac{4\pi}{3}R_{tot}^3 = \frac{4\pi}{3}R^3 + \frac{fN_k}{\rho_k} \tag{S1}$$

where R_{tot} is an expected radius of the whole object, N_k is the number of Kuhn segments per grafted chain (arm) and ρ_k is the number density of the Kuhn segments. The overall radius R_{tot} is a sum of the radius of the impenetrable layer, $h_{\rm dry}$, the radius R and half of the interpentrable region, $h_{\rm inter}$:

$$R_{tot} = R + h_{\rm dry} + h_{\rm inter}/2. \tag{S2}$$

The model solves equation for an unknown parameter $n_{\rm inter}$, which denotes the number of Kuhn segments in the interpenetration layer (for more details see the Supplementary Information in ref. [9]). The main parameter of the solution, the so-called overcrowding parameter x was formulated as:

$$x = \frac{f}{\pi\rho_k l_k^2[R^3 + 3fN_k/(4\pi\rho_k)]^{1/3}} \tag{S3}$$

where l_k is the Kuhn length. In the derivation, the prefactor 4/3 which appears in eq. S1 was omitted. Moreover, in order to achieve the final expression of eq. S3, one must assume a Gaussian conformation of the chains in the interpenetration layer. Alternatively, the eq. S3 can be reformulated as:

$$x = \frac{f}{\frac{\pi l_k^2}{\nu_0}[R^3 + 3fN_k\nu_0/(4\pi)]^{1/3}} \tag{S4}$$

where ν_0 is the volume of the Kuhn monomer. [10]

Without doing the above-mentioned assumptions, we can rely on the physical meaning of the overcrowding parameter. The quantity x can be defined as a ratio of the actual number of monomers in a single star, within a distance R', $N_{\rm intra,mers}(R')$, to the number of monomers that would occupy the same volume in an unperturbed melt, $N_{\rm ideal,mers}(R')$. By choosing the radius of gyration Rg as a characteritic radius of the spherical volume for the definition of x, i.e. $R' = Rg$, we obtain:

$$x = \frac{f}{\frac{4\pi\rho_n Rg^3}{3N_{\rm arm}}} = \frac{N_{\rm intra,mers}(R')}{N_{\rm ideal,mers}(R')} = \frac{fN_{\rm arm}}{4/3\pi\rho_N Rg^3} \tag{S5}$$

where $N_{\rm arm}$ is the number of monomers per arm and ρ_N the monomer number density. Note that N_k and ρ_k, instead of $N_{\rm arm}$ and ρ_N, can be also used in the above definition without a significant change in the value of x parameter. We opt for the monomer-based quantities as these can be easily calculated from the atomistic simulations and also they can be found in the literature. The overcrowding parameter defined by using eq. S3 and eq. S5 is plotted in Fig. S5 and listed in Tab. S1.

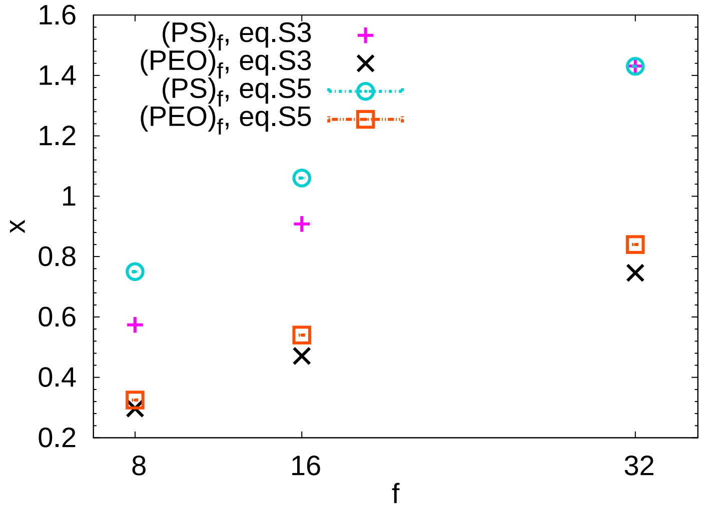

FIG. S5. Overcrowding parameter defined by using eq. S3 and eq. S5 as a function of functionality, f, of the stars.

VI. ADDITIONAL DATA RELATED TO THE ESTIMATION OF THE BRUSH THICKNESS

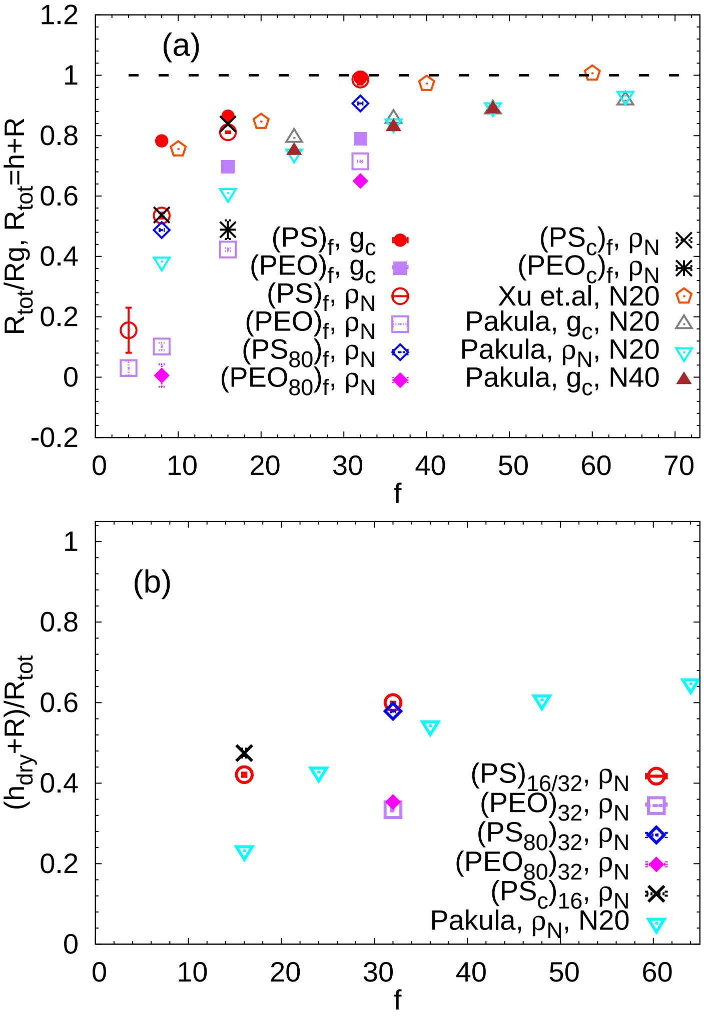

FIG. S6. Rescaled (a) total brush thickness and (b) thickness of the impenetrable region as a function of functionality f. The published data were extracted from ref. [7] and ref. [11].

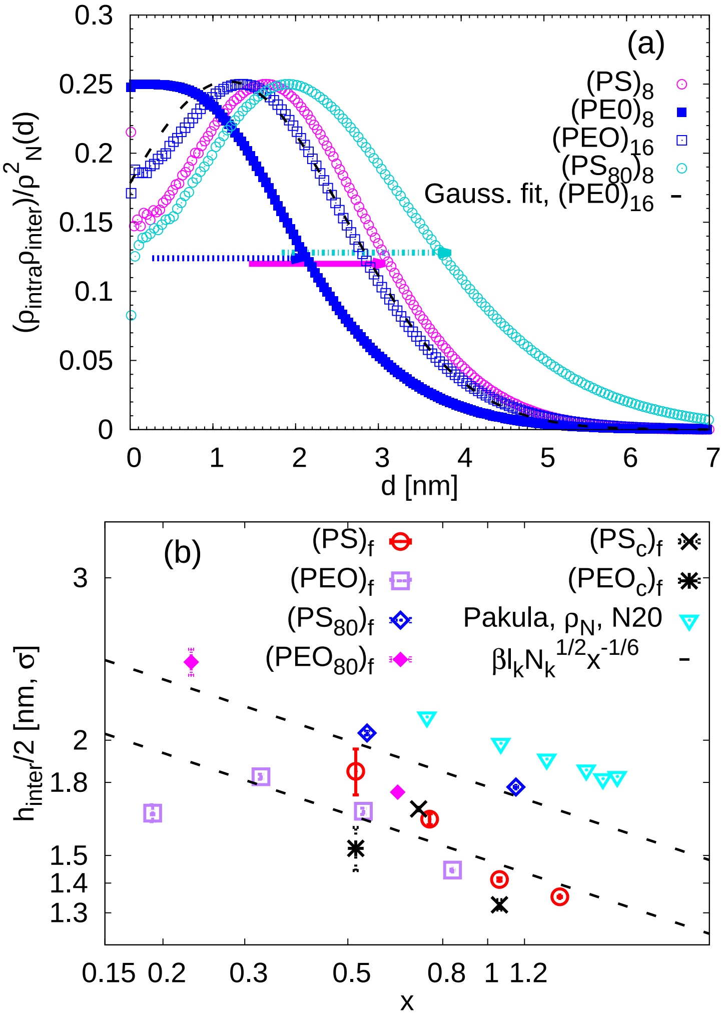

FIG. S7. (a) The product of intra- and inter-molecular density profile used for the estimation of $h = h_{\rm inter}/2 + h_{\rm dry}$. The dashed line represents a Gaussian fit and the arrows, starting at $d = h$, indicate the length equal to $h_{\rm inter}/2$ for each system with the corresponding color scheme. $h_{\rm inter}/2$ is defined as one half of the full width at half-maximum of the function $\rho_{\rm intra}.\rho_{\rm inter}$. (b) Thickness of the interpenetration layer $h_{\rm inter}$ as a function of the overcrowding parameter, x. The dashed lines represent the theoretical prediction of Kapnistos et al. [12]. The upper line corresponds to $\beta = 0.34$ and the bottom line to $\beta = 0.4$, using the values of l_k and N_k corresponding to the polystyrene stars.

REFERENCES

[1] Eirini Gkolfi, Petra Bačová, and Vagelis Harmandaris, "Size and shape characteristics of polystyrene and poly(ethylene oxide) star polymer melts studied by atomistic simulations," Macromolecular Theory and Simulations **30**, 2170001 (2021).

[2] Berk Hess, Carsten Kutzner, David van der Spoel, and Erik Lindahl, "Gromacs 4: Algorithms for highly efficient, load-balanced, and scalable molecular simulation," Journal of Chemical Theory and Computation **4**, 435–447 (2008).

[3] Daoud, M. and Cotton, J.P., "Star shaped polymers : a model for the conformation and its concentration dependence," J. Phys. France **43**, 531–538 (1982).

[4] T.M. Birshtein and E.B. Zhulina, "Conformations of star-branched macromolecules," Polymer **25**, 1453–1461 (1984).

[5] T.M Birshtein, E.B Zhulina, and O.V Borisov, "Temperature-concentration diagram for a solution of star-branched macromolecules," Polymer **27**, 1078–1086 (1986).

[6] Kyle J. Johnson, Emmanouil Glynos, Georgios Sakellariou, and Peter Green, "Dynamics of star-shaped polystyrene molecules: From arm retraction to cooperativity," Macromolecules **49**, 5669–5676 (2016).

[7] T. Pakula, "Static and dynamic properties of computer simulated melts of multiarm polymer stars," Computational and Theoretical Polymer Science **8**, 21 – 30 (1998).

[8] Christos N. Likos, "Effective interactions in soft condensed matter physics," Physics Reports **348**, 267–439 (2001).

[9] Jiarul Midya, Michael Rubinstein, Sanat K. Kumar, and Arash Nikoubashman, "Structure of polymer-grafted nanoparticle

melts," ACS Nano **14**, 15505–15516 (2020).
[10] Daniele Parisi, Eileen Buenning, Nikolaos Kalafatakis, Leo Gury, Brian C. Benicewicz, Mario Gauthier, Michel Cloitre, Michael Rubinstein, Sanat K. Kumar, and Dimitris Vlassopoulos, "Universal polymeric-to-colloidal transition in melts of hairy nanoparticles," ACS Nano **15**, 16697–16708 (2021).
[11] Xiaolei Xu and Jizhong Chen, "Effect of functionality on unentangled star polymers at equilibrium and under shear flow," The Journal of Chemical Physics **144**, 244905 (2016).
[12] Michael Kapnistos, Alexander N. Semenov, Dimitris Vlassopoulos, and Jacques Roovers, "Viscoelastic response of hyperstar polymers in the linear regime," The Journal of Chemical Physics **111**, 1753–1759 (1999).
